# Sex differences in single and combined cardiovascular medication use

**DOI:** 10.1016/j.ahjo.2026.100866

**Published:** 2026-08-22

**Authors:** T.C.X. Duk, A. Uijl, P.A.J. Kiss, E. Smits, D.E. Grobbee, S.A.E. Peters

**Affiliations:** a Julius Center for Health Sciences and Primary Care, Global Public Health and Bioethics, University Medical Center Utrecht, Utrecht University, Utrecht, the Netherlands; b Department of Clinical Science and Education, Södersjukhuset, Karolinska Institutet, Stockholm, Sweden; c PHARMO Institute for Drug Outcomes Research, Utrecht, the Netherlands; d The George Institute for Global Health, School of Public Health, Imperial College London, London, United Kingdom; e The George Institute for Global Health, University of New South Wales, Sydney, New South Wales, Australia

**Keywords:** Cardiovascular drugs, Cardiovascular disease, Sex differences, Drug combinations, Polypharmacy, Drug utilization

## Abstract

**Introduction:**

Drug therapy is a key component in the prevention and management of cardiovascular disease (CVD). In this study, we assess sex differences in the use of single and combined cardiovascular medication.

**Methods:**

We analyzed data from individuals ≥40 years old between 2010 and 2020 in the PHARMO Data Network, which included data from out-patient pharmacies and primary care across the Netherlands. Included individuals were either at high-risk for CVD or were previously diagnosed with CVD. Dispensed cardiovascular medications were categorized into ten medication classes, based on their active substances. Concomitant medication was defined as medication dispensed in the same semester. Sex-specific dispense patterns and women-to-men prevalence ratios (PRs) were calculated for groups stratified by age and CVD status.

**Results:**

In total, 53,110 individuals at high-risk for CVD (36% women) and 71,996 individuals diagnosed with CVD (45% women) were included. Fifty out of 645 drug combinations had a median prevalence of ≥1%. Combinations of five or more drugs were more frequent in men compared to women, regardless of age and CVD status (range prevalence difference between sexes: 2–5%). Specifically, three combinations of five or more drugs were more often dispensed to men than women (PR range: 0.5–0.7).

**Conclusion:**

This study highlights sex differences in the number and types of dispensed combined cardiovascular medication, with dispenses of five or more cardiovascular drugs being more common in men than women. Age, sex, and CVD status contributed to differences in specific dispensed combinations.

## 1 Introduction

Cardiovascular diseases (CVD) are a major public health concern [1]. One of the pillars for prevention and management of CVD in those at high-risk or with established CVD is cardiovascular pharmacotherapy [2]. Previous longitudinal studies showed that in the general population in Europe and North America, antihypertensive drugs were prescribed in 22% of adults, antithrombotics in 10% of adults, and statins in 28% of adults [3], [4], [5]. For secondary prevention, antihypertensives were prescribed in 70% of adults and antiplatelets and statins were used in over half of the adults [6].

Previous studies on sex differences in cardiovascular pharmacotherapy have mainly focused on individual therapies [7], [8], [9]. A meta-analysis showed that women were less likely than men to be prescribed a statin in primary care. While the use of antihypertensives was similar between the sexes, there were differences in specific medication classes, with women being less likely to be prescribed an angiotensin-converting enzyme inhibitor (ACEi), but more likely to be prescribed a diuretic [9].

CVD medications are widely used in combinations, with the simultaneous use of five or more drugs or pharmacologically active substances being described as polypharmacy [10], [11]. In one study prescribed CVD medication lead to polypharmacy in 18% of adults [11]. Still, it remains unclear what different cardiovascular drug combinations are used by women and men in primary care.

Therefore, in this study, we assessed sex differences in cardiovascular medication dispense combinations in a routine care setting.

## 2 Methods

### 2.1 Data source

This study used electronic health records from the PHARMO Data Network on more than ten million people from 1998 onwards. The PHARMO Data Network consists of data from different primary and secondary healthcare settings in the Netherlands, including general practitioner (GP) data (∼20% of the Dutch population), out-patient pharmacy data (∼25% of the Dutch population), and hospital data (80% of hospitals in the Netherlands) [12]. Data from these individual databases is linked using probabilistic linkage [13]. The PHARMO Data Network was shown to be an accurate representation of the Dutch population in demographic characteristics, GP prescriptions, and GP diagnoses [14].

### 2.2 Study population

Individuals 40 years old and above who were part of the PHARMO GP data between January 2010 and December 2020, and either at high-risk for CVD or diagnosed with CVD, were included. CVD risk score was determined per annual semester (i.e., January–June or July–December) using the SCORE2 and SCORE2-OP algorithms [15], [16]. SCORE2 was applied to individuals 40–69 years old and SCORE2-OP to individuals 70 years old and older respectively. Based on the guidelines by the Dutch College of General Practitioners [17], individuals were included in the study as high-risk for CVD, if: 1) their risk score was classified as very high-risk, 2) their estimated glomerular filtration rate (eGFR) was <29 mL/min/1.73m^2^, or 3) they were diagnosed with diabetes mellitus (International Classification of Primary Care (ICPC) code: T90 and all subcodes) and were (3a) a current smoker, (3b) had a systolic blood pressure (SBP) ≥180 mmHg, or (3c) a total cholesterol of >8 mmol/L. Patients diagnosed with CVD were identified based on the ICPC codes for CVD (K74-K80, K83, K84, K89, K90, K92, K99) or presence of a pacemaker or defibrillator (A89.01) (Supplementary Table 1) [18]. The index date was defined as the date when someone would be classified as either high-risk for or diagnosed with CVD. The last measurement date used in the risk score calculation was used as the index date for people with a very high-risk score. The index date for high-risk individuals with a diabetes mellitus diagnosis was defined as the earliest date when a threshold value was met for SBP, total cholesterol, or smoking. If the diagnosis of diabetes mellitus occurred within five years after the threshold was first met, that diagnosis date was used as the index date instead.

People were included if they had at least one recorded drug dispense from the first full annual semester after the index date onwards, in the out-patient pharmacy data. Individuals at high-risk for CVD were considered diagnosed with CVD from the start of the first semester after receiving a CVD diagnosis. A flowchart of the selection of the study population is shown in Supplementary Fig. 1, and a graph of the study population per semester is shown in Supplementary Fig. 2.

### 2.3 Study variables

Demographic data included sex, birth year, and social economic status (SES). SES classification was based on the 2014 score given by the Netherlands Institute for Social Research based on four-digit postal code.

Baseline measurements for SBP, diastolic blood pressure (DBP), body mass index (BMI), total cholesterol, high density lipoprotein cholesterol (HDL-c), smoking, and eGFR performed in the six months before the index date were extracted from the GP examination data, and implausible values were removed (Supplementary Table 2). Creatinine values were also used to calculate eGFR values using the CKD-EPI equation in case of missing eGFR [19].

Medical history of CVD (i.e., arrhythmia, coronary heart disease (CHD), heart failure, peripheral artery disease, stroke, or another CVD), atherosclerosis, CKD, or diabetes mellitus was defined as a diagnosis with ICPC code during GP consultation before or during the index semester (Supplementary Table 1). Hypertension was defined by either a recorded ICPC code before or during the index semester, or an average recorded SBP/DBP of ≥140/90 mmHg in the six months before the index date.

Dispensed cardiovascular drugs were recorded from the start of the first new annual semester after the index date and contained information on dispense date, amount dispensed, and complete WHO Drug Anatomical Therapeutic Chemical (ATC) code [20].

### 2.4 Dispensed medication

The study outcomes are the number and types of dispensed cardiovascular medication. As some drugs contain multiple active substances, drugs were categorized and counted by active substance [11]. Active substances were categorized by ATC code into ten cardiovascular drug type categories: antithrombotic drugs, digoxin, sodium-glucose cotransporter 2 inhibitors (SGLT2i), antihypertensives (beta blockers (BB), angiotensin II receptor antagonists (ARB), ACEi, calcium channel blockers (CCB), diuretics), statins, and other lipid-level modifiers (Supplementary Table 3). Unique drug types dispensed in the same annual semester were considered concomitant dispenses. The time interval of one semester was based on the number of drugs per dispense, so the majority of dispenses would not be missed in a semester that they would be used (Supplementary Fig. 3).

### 2.5 Statistical analysis

Statistical analysis was performed with R Statistical Software v4.2.1 [21]. Supplementary Fig. 4 shows a flowchart of the statistical analysis.

Baseline characteristics were calculated by sex and age (<70 or ≥70 years old). Missing data were not imputed as they were not used in the statistical analyses. Absence of medical history, drug dispenses, or smoking status (68% of records) was considered absence of that specific condition.

The sex-specific prevalence for the number and types of dispensed cardiovascular medication was calculated per annual semester, stratified by age (<70 or ≥70 years old) and CVD status (high-risk CVD or CVD diagnosed). Results were summarized over all semesters. Only drugs and drug combinations with a median prevalence ≥1% over the complete period were included to allow for stable comparisons. Rescaling to 100% was done after excluding less common combinations and people without dispenses to allow for comprehensive comparison of the most common combinations in each group. In a sensitivity analysis, the prevalences of dispensed drug and drug combination types were determined separately for patients diagnosed with CHD, as this was the most prevalent CVD subtype.

Women to men prevalence ratios (PRs) were calculated to assess sex differences. In case no dispensing was recorded in one of the sexes, an event count of 0.5 was used in the prevalence calculation to ensure all prevalence ratios could be calculated [22]. A ratio lower than 0.8 or above 1.2 was considered to indicate a clinically meaningful sex difference.

## 3 Results

### 3.1 Baseline characteristics

A total of 125,106 individuals were included: 2936 women and 12,417 men <70 years old at high-risk for CVD; 15,961 women and 21,796 men ≥70 years old at high-risk for CVD; 15,284 women and 24,534 men <70 years old with a CVD diagnosis; and 17,427 women and 14,751 men ≥70 years old with a CVD diagnosis (Table 1). The median age per group was 66 [IQR: 7], 66 [IQR: 5], 83 [IQR: 5], 77 [IQR: 7], 61 [IQR: 11], 61 [IQR: 10], 77 [IQR: 9], and 75 [IQR: 8] years respectively. Among individuals with a CVD diagnosis, coronary heart disease was diagnosed in 30% of women and 41% of men <70 years old and 23% of women and 29% of men ≥70 years old.

**Table 1:** Baseline characteristics stratified by sex, age, and CVD status.

	high-risk for CVD				CVD diagnosed				Missing^a^
	<70 years old		≥70 years old		<70 years old		≥70 years old		
	Women	Men	Women	Men	Women	Men	Women	Men	
n	2936	12,417	15,961	21,796	15,284	24,534	17,427	14,751	
Age (years)^c^	66 [61, 68]	66 [63, 68]	83 [80, 85]	77 [74, 81]	61 [55, 66]	61 [55, 65]	77 [73, 82]	75 [72, 80]	0
Socioeconomic status^a^									0.1
Low	36	33	36	33	34	32	35	33	
Middle	41	41	42	42	40	41	41	41	
High	24	26	22	25	26	27	24	26	

Risk factors									
Systolic blood pressure (mmHg)^b^	154.6 (23.2)	154.1 (18.0)	150.6 (18.3)	147.1 (17.2)	141.3 (19.8)	141.2 (19.3)	144.6 (19.2)	140.8 (19.4)	30.8
Diastolic blood pressure (mmHg)^b^	85.1 (10.9)	85.9 (10.0)	78.8 (9.3)	78.9 (9.6)	82.5 (10.6)	83.3 (10.8)	78.7 (10.0)	77.8 (10.6)	31
Body mass index (kg/m^2^)^b^	29.5 (5.8)	28.9 (4.3)	28.0 (4.9)	27.6 (3.8)	29.5 (6.4)	28.9 (4.6)	28.4 (5.2)	27.7 (4.1)	61.3
Smoking^a^	68	39	8	8	9	8	3	3	0
Total cholesterol (mmol/L)^b^	5.8 (1.3)	5.5 (1.2)	5.3 (1.2)	4.8 (1.1)	5.5 (1.2)	5.1 (1.2)	5.2 (1.1)	4.5 (1.0)	45.1
High density lipoprotein (mmol/L)^b^	1.2 (0.3)	1.1 (0.3)	1.4 (0.4)	1.2 (0.3)	1.5 (0.4)	1.2 (0.3)	1.5 (0.4)	1.3 (0.4)	45.2
HbA1c (mmol/mol)^b^	51.6 (14.0)	50.6 (14.6)	49.1 (10.6)	48.8 (11.0)	49.9 (13.4)	50.1 (13.3)	49.5 (10.5)	50.2 (11.6)	76.1
eGFR (mL/min/1.73m^2^)^b^	66.2 (18.2)	69.5 (15.4)	59.2 (15.6)	62.9 (14.8)	68.5 (14.7)	70.0 (14.9)	61.1 (14.5)	62.1 (14.5)	44.2
Cardiovascular Risk score^b^	9.8 (3.1)	11.3 (2.2)	18.2 (3.9)	18.6 (3.8)	4.7 (2.2)	6.6 (2.4)	11.4 (4.2)	14.3 (4.2)	48.6
Chronic kidney disease^a^	6	3	9	5	3	2	7	5	0
Diabetes mellitus^a^	36	16	12	10	4	4	3	3	0
Hypertension^a^	44	45	24	22	21	19	14	19	0
Atherosclerosis^a^	5	7	8	9	6	7	9	9	0

CVD history^a^									
Coronary heart disease	0	0	0	0	30	41	23	29	0
Arrhythmia	0	0	0	0	24	19	26	23	0
Stroke	0	0	0	0	20	17	21	18	0
Heart failure	0	0	0	0	4	4	12	10	0
Peripheral arterial disease	0	0	0	0	12	10	9	8	0
Other CVD	3	3	2	2	13	12	14	16	0

Cardiovascular medication history									
Total unique dispensed drugs^c^	2 [1, 3]	2 [1, 3]	3 [2, 4]	3 [1, 4]	3 [1, 4]	3 [2, 4]	3 [2, 4]	3 [2, 5]	0
Number of concomitant active substances^a^									0
0	11	13	7	10	12	10	7	7	
1	22	23	17	17	15	9	9	8	
2	24	23	23	21	20	17	17	16	
3	20	19	23	21	19	20	21	20	
4	14	13	18	17	18	24	22	23	
≥5	9	10	14	14	16	20	24	25	
Antihyperlipidemia^a^	56	52	45	54	56	68	54	65	0
Antihypertensives^a^	75	73	85	78	73	75	83	80	0
Antithrombotics^a^	21	27	44	52	64	76	75	83	0
Other cardiovascular medication^a^	1	1	3	2	2	3	7	6	0

a (%).

b (Mean (standard deviation)).

c (Median [Q1–Q3]).

The median number of dispensed drugs was two for people <70 years old at high-risk CVD, three for people ≥70 years old at high-risk CVD, and three for women and men diagnosed with CVD.

### 3.2 Medication quantity

**Fig. 1:**
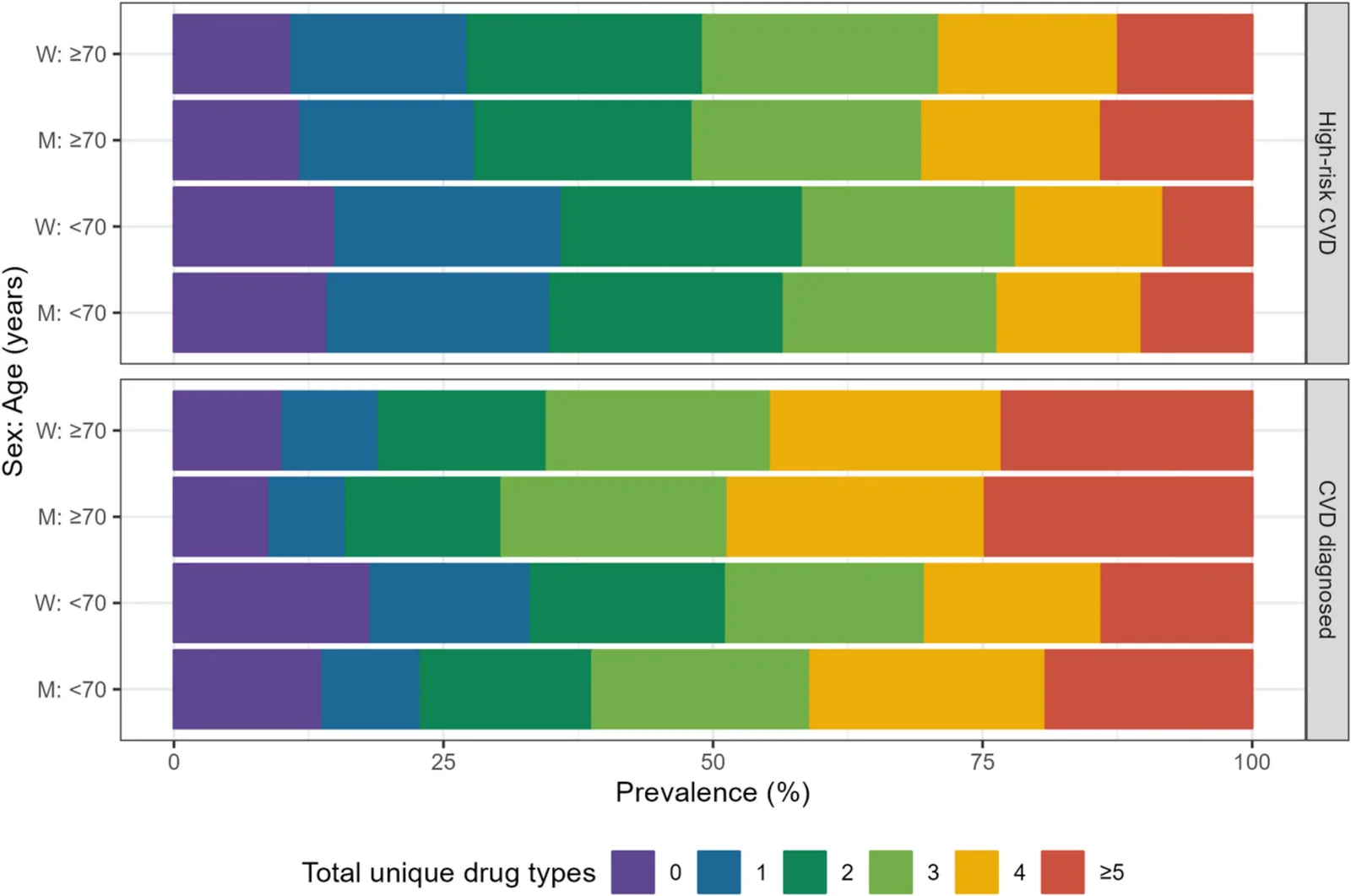
Proportion of median number of dispensed CVD drugs from 2010 to 2020, scaled to 100%.

The proportion of dispensed drug types was broadly constant between 2010 and 2020 (Supplementary Fig. 5). Among individuals at high-risk for CVD, 15% of women and 14% of men <70 years old, and 11% of women and 12% of men ≥70 years old had no recorded dispenses (Fig. 1). Five or more drugs were dispensed to 8% of women and 10% of men <70 years old, and 12% of women and 14% of men ≥70 years old.

Among individuals diagnosed with CVD, 18% of women and 14% of men <70 years old and 10% of women and 9% of men ≥70 years old had no recorded dispenses. Five or more drugs were dispensed to 14% of women and 19% of men <70 years old and 23% of women and 25% of men ≥70 years old.

### 3.3 Medication combinations

**Fig. 2:**
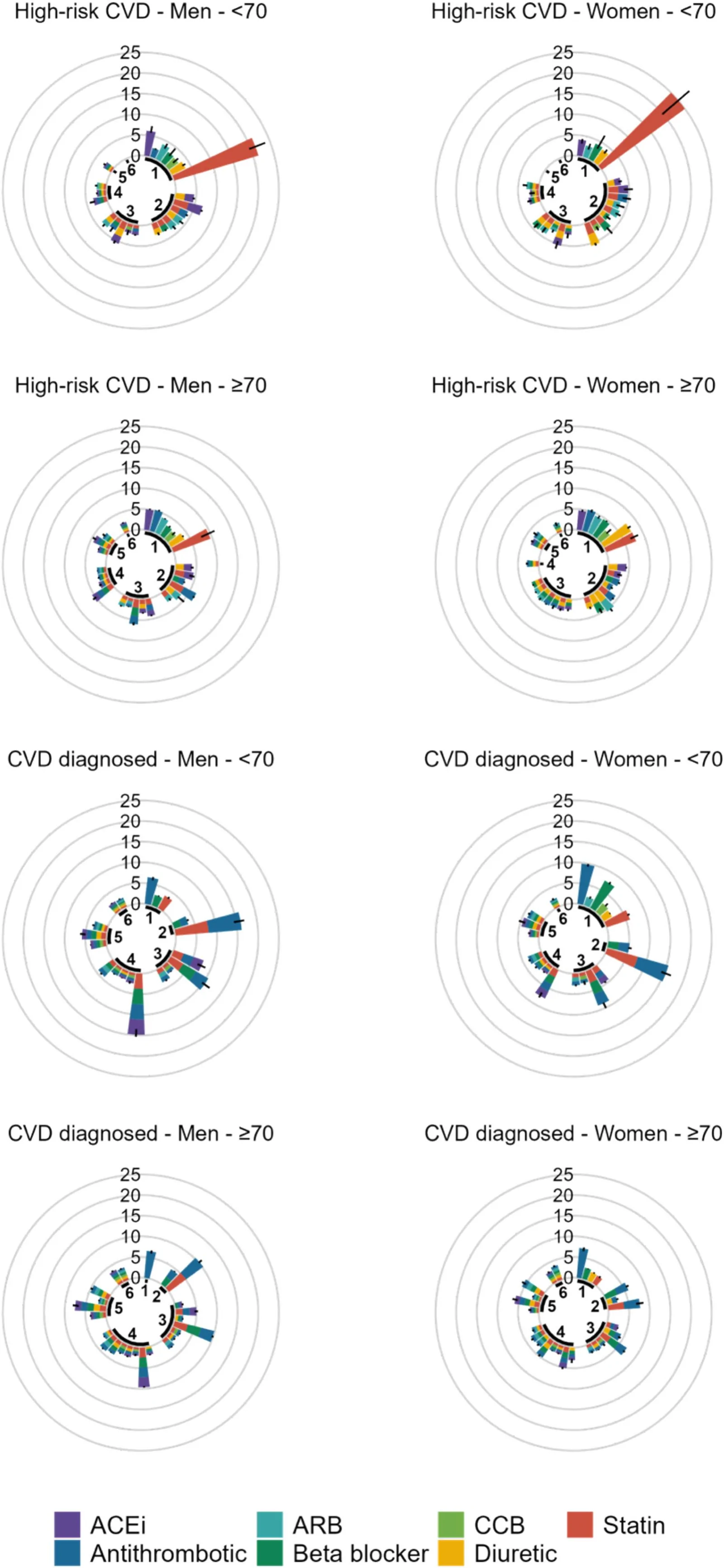
Median prevalence of single and combined dispensed drugs by sex, age, and CVD status. Median prevalence was taken from 2010 to 2020. Error bars indicate the first and third quartile.

In total, there were 645 distinct single and combined drug dispenses, of which fifty had a median prevalence of ≥1% in at least one comparison group. Out of these fifty combinations, seven were single dispensed drugs, 37 were combinations of two to four drugs, and six were combinations of five or six drugs. The prevalences of these drug combinations can be found in Fig. 2 and Table 2. In those at high-risk for CVD, these combinations comprising more than one drug type were dispensed to 30% of women and 27% of men <70 years old, and 27% of women and 32% of men ≥70 years old. In those diagnosed with CVD, these combinations were dispensed to 33% of women and 48% of men <70 years old, and 44% of women and 55% of men ≥70 years old.

**Table 2:** Scaled median prevalence (2010–2020) and [IQR] for the dispensed drug combinations, stratified by sex, age, and CVD status. Only the drug combinations that had an unscaled prevalence of ≥1% are included.

Drug combination	n drugs	High-risk for CVD				CVD diagnosed			
		<70 years old		≥70 years old		<70 years old		≥70 years old	
		Women	Men	Women	Men	Women	Men	Women	Men
ACEi	1	4 [1.2]	6.1 [1.7]	4.7 [0.5]	5 [0.3]				
Antithrombotic	1		2.2 [0.4]	5.3 [0.7]	5.2 [0.4]	9.8 [0.7]	6.5 [0.8]	7.2 [0.9]	6.6 [0.8]
ARB	1	2.9 [1.5]	3.9 [1.3]	4.5 [0.8]	4 [0.3]	2.2 [0.6]			
Beta blocker	1	4.1 [3.6]	3 [2.3]	4.7 [0.3]	2.9 [0.5]	7.5 [0.6]	2.7 [0.3]	2.7 [0.2]	
CCB	1		2.8 [1.6]	2.8 [1.5]	2.5 [0.9]	2.6 [0.9]			
Diuretic	1	3.7 [1.1]	3.6 [0.9]	7.6 [0.9]	3.7 [0.4]	2.3 [0.2]		2.2 [0.3]	
Statin	1	25 [8.6]	21.3 [4.1]	7.7 [2.1]	9.5 [3.5]	5.9 [0.6]	3.5 [0.5]	2.2 [0.5]	
ACEi - Diuretic	2	3.1 [0.8]	4.5 [0.5]	4.3 [0.3]	3.9 [0.6]				
ACEi - Statin	2	4.8 [2.1]	7 [0.5]	3.1 [1.1]	4.5 [1.2]				
Antithrombotic - Beta blocker	2			3.3 [0.5]	2.8 [0.3]	5.2 [0.8]	3.6 [0.5]	5.9 [0.7]	4.4 [0.2]
Antithrombotic - Diuretic	2			2.3 [0.5]				2.5 [0.5]	
Antithrombotic - Statin	2	4.7 [2]	4.1 [0.5]	4.2 [0.4]	6.7 [0.4]	15.4 [2.5]	16.1 [2.5]	7.7 [1.8]	10.3 [0.9]
ARB - Diuretic	2	3.3 [2]	3.8 [1.1]	5.6 [0.7]	3.7 [1.9]				
ARB - Statin	2	3.8 [1.3]	3.5 [1]		2.7 [0.7]				
Beta blocker - Diuretic	2	2.1 [1.2]		4.7 [1.2]					
Beta blocker - Statin	2	3.9 [1.9]	2.9 [0.4]						
CCB - Statin	2	2.5 [1]							
Diuretic - Statin	2	5.6 [0.6]	2.9 [0.7]	2.8 [0.6]					
ACEi - Antithrombotic - Beta blocker	3								1.8 [0.4]
ACEi - Antithrombotic - Statin	3		2.4 [0.3]		4.2 [0.4]	4.5 [0.4]	7.6 [2]	2.8 [0.2]	5 [0.8]
ACEi - Beta blocker - Diuretic	3	2.3 [1.2]		2.5 [0.5]					
ACEi - CCB - Statin	3		2.2 [1.3]						
ACEi - Diuretic - Statin	3	5.3 [1.9]	5.6 [0.7]	3 [0.6]	3.3 [0.4]				
Antithrombotic - Beta blocker - Diuretic	3			2.9 [0.9]				4.2 [0.6]	1.8 [0.3]
Antithrombotic - Beta blocker - Statin	3		3 [1.2]	3.2 [0.3]	6.1 [0.7]	8.8 [1.5]	10.5 [2.2]	7 [0.5]	9.9 [0.5]
Antithrombotic - CCB - Statin	3					2.6 [0.9]	2.3 [0.2]	2.1 [0.6]	2.3 [0.3]
Antithrombotic - Diuretic - Statin	3							2.4 [0.2]	1.8 [0.3]
ARB - Antithrombotic - Statin	3				2.2 [0.2]	2.9 [0.4]	3.4 [0.4]	2.1 [0.4]	2.7 [0.3]
ARB - Beta blocker - Diuretic	3	2.4 [0.7]		3.3 [0.6]					
ARB - Diuretic - Statin	3	4 [1.4]	3.6 [0.7]	2.9 [0.3]	2.6 [0.3]				
Beta blocker - Diuretic - Statin	3	4.3 [1.3]		2.6 [0.3]					
ACEi - Antithrombotic - Beta blocker - Diuretic	4							3.1 [0.9]	1.9 [0.3]
ACEi - Antithrombotic - Beta blocker - Statin	4		3.3 [1.2]		5.5 [0.9]	7.6 [1.3]	14.8 [1.8]	5.2 [0.5]	9.6 [0.3]
ACEi - Antithrombotic - CCB - Statin	4						2.6 [0.6]		2 [0.5]
ACEi - Antithrombotic - Diuretic - Statin	4				2.7 [0.6]	2.3 [0.3]	2.1 [0.1]	2.1 [0.3]	2.6 [0.3]
ACEi - Beta blocker - Diuretic - Statin	4	2.6 [1]	2.2 [0.3]						
ACEi - CCB - Diuretic - Statin	4	2.1 [1.3]							
Antithrombotic - Beta blocker - CCB - Statin	4						2.2 [0.2]	2.2 [0.2]	2.5 [0.3]
Antithrombotic - Beta blocker - Diuretic - Statin	4			2.8 [0.5]	2.2 [0.4]	2.8 [0.4]		4.8 [0.5]	3.6 [0.2]
ARB - Antithrombotic - Beta blocker - Diuretic	4							3.1 [0.5]	
ARB - Antithrombotic - Beta blocker - Statin	4				2.2 [0.1]	3.5 [0.4]	4.3 [0.3]	3.4 [0.6]	4.2 [0.3]
ARB - Antithrombotic - Diuretic - Statin	4							2.3 [0.4]	2.1 [0.2]
ARB - Beta blocker - Diuretic - Statin	4	3 [1.1]							
ARB - CCB - Diuretic - Statin	4		2.3 [0.5]						
ACEi - Antithrombotic - Beta blocker - CCB - Statin	5				2.5 [0.3]	2.4 [0.5]	3.8 [0.1]	2.1 [0.7]	3.7 [0.3]
ACEi - Antithrombotic - Beta blocker - Diuretic - Statin	5		2.2 [0.5]	3 [0.6]	4.3 [0.9]	4.5 [1.6]	5.8 [1.2]	6 [1.3]	7.5 [1.4]
ARB - Antithrombotic - Beta blocker - CCB - Statin	5							2 [0.3]	2.1 [0.3]
ARB - Antithrombotic - Beta blocker - Diuretic - Statin	5			3.3 [1]	2.8 [0.7]	3.9 [0.5]	3.3 [0.5]	5.6 [1.1]	4.7 [0.5]
ACEi - Antithrombotic - Beta blocker - CCB - Diuretic - Statin	6				2.5 [0.3]		2.6 [0.2]	3 [0.4]	3.6 [0.3]
ARB - Antithrombotic - Beta blocker - CCB - Diuretic - Statin	6			2.3 [0.9]		2.2 [0.5]	2.3 [0.2]	3.7 [0.4]	3.2 [0.3]

Only the combination statin – antithrombotic was found in all comparison groups. Nineteen combinations were exclusively dispensed to high-risk individuals. Of those, six were seen in all age and sex groups. Nine combinations were unique to individuals diagnosed with CVD, one (antithrombotic – statin) being present in all groups. Other combinations were only present in either men or women depending on CVD status: four combinations (BB – diuretic; ACEi – BB – diuretic; ARB – BB – diuretic; BB – diuretic – statin) were present in women and not in men at high-risk for CVD and one combination (ACEi – antithrombotic – CCB – statin) was dispensed to men and not women diagnosed with CVD. Two combinations were present solely in individuals <70 years old at high-risk for CVD, and four combinations were present solely in individuals ≥70 years old diagnosed with CVD.

A sensitivity analysis of patients diagnosed with coronary heart disease (CHD) can be found in Supplementary Fig. 6. Fourteen out of 27 drugs and drug combinations were present in all groups. The two drug combinations with the highest prevalence were ACEi – antithrombotic – BB – statin and antithrombotic – BB – statin. The former was present in 16% of women and 22% of men <70 years old and 11% of women and 17% of men ≥70 years old, and the latter was present in 14% of individuals <70 years old and 12% of women and 15% of men ≥70 years old.

### 3.4 Prevalence ratios medication

**Fig. 3:**
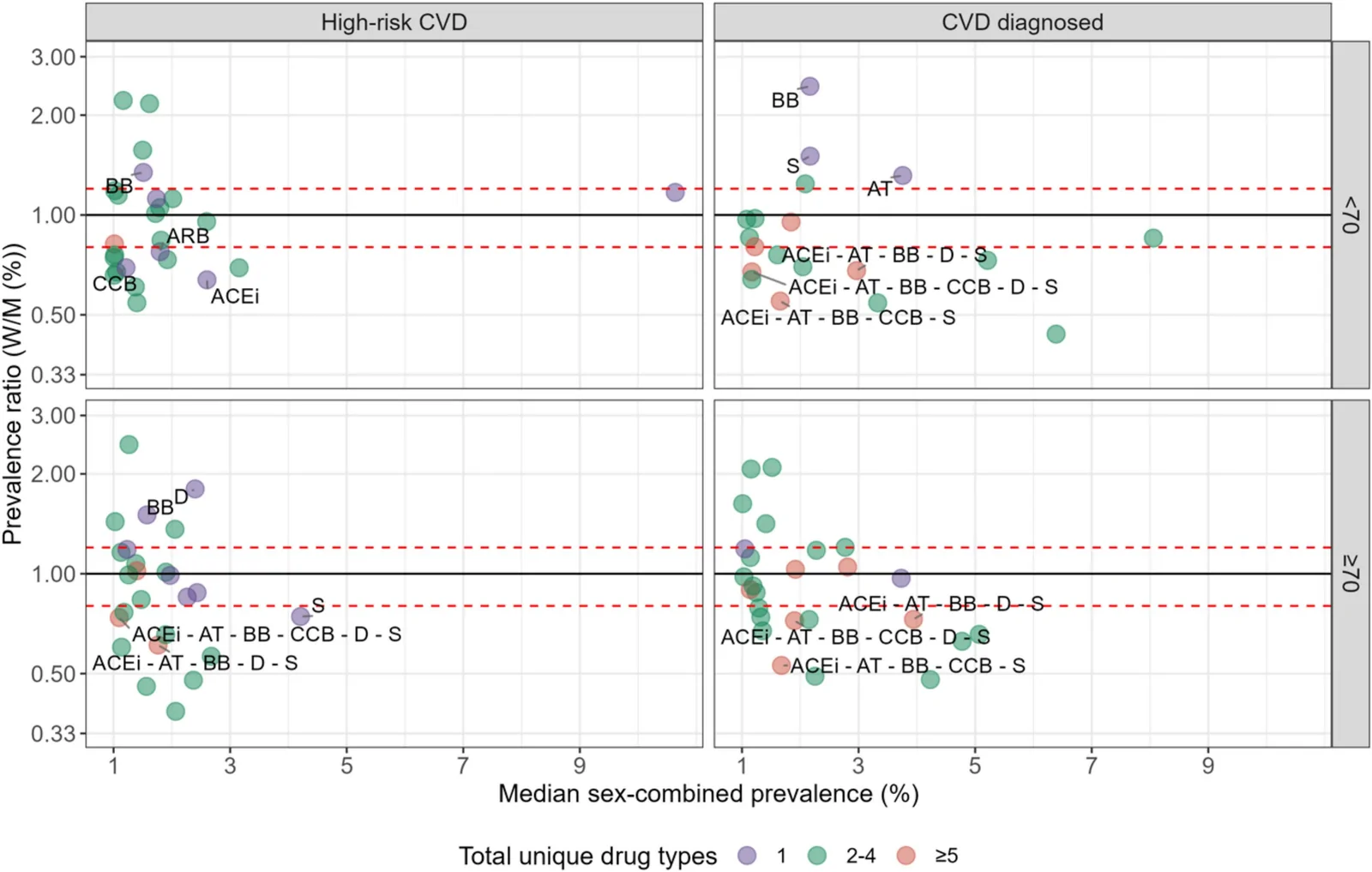
Women to-men prevalence ratios of drug dispenses. Only combinations with a median prevalence of ≥1% for women and men combined are included. The logarithmic y-axis shows the prevalence ratio, where a prevalence ratio >1 or <1 indicates a higher prevalence in women and men respectively. A clinical significance threshold of 20% is indicated. Single drugs and drug combinations of five or more drugs that passed the significance threshold are annotated.

Women to men PRs of dispensed medications with a sex-combined prevalence of ≥1% are shown in Fig. 3 and Supplementary Table 4. All combinations of five or more drugs with a clinically significant difference favored men (PR of <0.8) (ACEi – antithrombotic – BB – diuretic – statin, ACEi – antithrombotic – BB – CCB – diuretic – statin, and ACEi – antithrombotic – BB – CCB – statin). Single beta blockers were more frequently dispensed to women (PR ≥1.2) in all groups except individuals ≥70 years old diagnosed with CVD. Single CCBs, ARBs, and ACEis were more frequently dispensed to men <70 years old at high-risk for CVD and statins were more frequently dispensed to men ≥70 years old at high-risk for CVD.

## 4 Discussion

This study assessed sex differences in dispensed cardiovascular medication among 125,106 individuals at high-risk for CVD or diagnosed with CVD. Out of 645 single drugs and drug combinations, fifty had a median prevalence of ≥1%, and the drug combinations with a prevalence ≥1% were dispensed to 40% of individuals overall. Polypharmacy was more common in men than women. Women-over-men PRs indicated that three poly-pharmaceutical combinations were more common in men than women: ACEi – antithrombotic – BB – diuretic – statin, ACEi – antithrombotic – BB – CCB – diuretic – statin, and ACEi – antithrombotic – BB – CCB – statin.

### 4.1 Sex differences in polypharmacy

Polypharmacy in CVD medication was more common in men than in women, regardless of age or CVD history. Specifically, differences were most pronounced in people <70 years old diagnosed with CVD. This is in line with a study that showed an attenuation with older age in the simultaneous use of cardiovascular medications for secondary prevention of CHD [7]. Another study found that both older age and number of comorbidities were the strongest predictors for polypharmacy overall [23]. However, one-to-one comparisons of risk factors for CVD polypharmacy remain complicated as few studies have been conducted on the risk factors between CVD medication and polypharmacy.

Contrastingly, studies investigating polypharmacy including both cardiovascular and non-cardiovascular medication showed that polypharmacy was more common in women than men [24], [25]. This could be explained by the higher use of non-cardiovascular medication in these women compared to men. This was also shown in another study in Swiss primary care, which reported that polypharmacy was more prevalent in women, except for cardiovascular medication, which was more prevalent in men [26]. Similarly, a Spanish study in people ≥65 years old also showed that polypharmacy was more common in women than men, but men were more often prescribed antihypertensives and statins [27].

### 4.2 Sex differences in cardiovascular medication by CVD status

CVD and CVRM guidelines do not provide sex-specific treatment combinations [17], [28], [29], [30], [31], [32]. This is due to a lack of clinical evidence generated by randomized controlled trials (RCTs), as women have been largely underrepresented in RCTs on cardiovascular medication and limited sex-stratified results on safety and efficacy are available [33]. Yet, we found sex differences in the types of drugs dispensed, especially among those at high-risk for CVD, regardless of age. Although the risk scores with SCORE2 did not differ much between women and men, the women in the age category ≥70 years old were, on average, older than men, which could have influenced the dispensed drug types [7]. CVRM guidelines also recommend adjustment of medication strategy in case of decreased kidney function [17], [34], which could partly explain the sex differences in drug combinations, as more women than men had a CKD diagnosis. Another possible reason for differences in drug combinations, could be the sex differences in reported adverse drug reactions [33], [35]. Side effects are more often reported for women for all types of CVD medication [33], which could lead to changes in drug treatment to alleviate or avoid these effects. Lastly, the differences between women and men in pharmacokinetics and pharmacodynamics of CVD medication could also underlie the decision for different drugs or drug combinations to treat CVD [35]. For example, the antihypertensive response and heart failure outcomes were better in women using ARBs compared to ACEis, while for men it was better when using ACEis [35], [36].

### 4.3 Sex differences in cardiovascular medication in CHD patients

The drug combinations found in patients diagnosed with CVD were more concentrated on specific drug combinations, as combination therapy is often recommended for CVDs [28], [29], [30], [31], [32]. CHD was the most common CVD diagnosis at baseline, as it was present in approximately a third of the people diagnosed with CVD, which was also reflected in the dispensed drug types. A European study looking at the combined drug use in CHD, found that 64% of CHD patients were using at least an antiplatelet, a statin, and an antihypertensive [37], corresponding to the most common combinations we found. Sex differences in the use of CHD drug therapy were reported by multiple studies: women were less often prescribed combination therapy of three or four drug types [7], [38]. In this study, the largest prevalence difference in dispensed combination was for the combined use of an ACEi, antithrombotic, BB, and statin (6%), while the sex differences between triple drug combinations remained relatively small (0–4%). Discrepancies with previous studies could be due to the differences in age inclusion criteria, which had a minimum age of 18 and 25 for the aforementioned studies, and differences tended to decrease with age [7], [38].

### 4.4 Strengths and limitations

This study provides a comprehensive overview of sex differences in cardiovascular medication dispensing. We used a large electronic health records database in a routine care setting in the Netherlands, that is representative of the Dutch population [14]. Although in-patient pharmacies were not included in this study, dispenses from in-patient pharmacies are commonly continued in out-patient pharmacies. As we determined combinations per semester, the size of the interval allowed for capturing repeat medication in out-patient pharmacies. It is thus likely they will resemble the dispense patterns described in this paper.

This study also has some limitations. First, overestimation of concomitant drug combinations is likely, as concomitant drugs were defined by dispense date within the same annual semester. Depending on dose, drug episodes could be disjointed. However, many of these cardiovascular drugs are prescribed to be taken continuously over time. In addition, the half-yearly periods were used to maximize the likelihood of capturing concomitant medication. Second, as we already included all major CVD medication classes, drug dose was not considered. Third, missing data was handled by taking half-yearly periods for the risk score calculation. We assumed that the out-patient pharmacy data fully captured dispensed medication for all individuals. Fourth, misclassification could have occurred as CVD history data was available from 2010 onwards, diluting the differences in medication between the two CVD history groups.

## 5 Conclusion

Women experience polypharmacy less often than men, regardless of age or CVD history. Many identical medication combinations were dispensed in the population, with variations by CVD history, sex, and age.

## CRediT authorship contribution statement

**T.C.X. Duk:** Writing – review & editing, Writing – original draft, Visualization, Methodology, Formal analysis, Data curation, Conceptualization. **A. Uijl:** Writing – review & editing, Supervision, Methodology, Conceptualization. **P.A.J. Kiss:** Writing – review & editing, Data curation. **E. Smits:** Writing – review & editing, Resources, Data curation. **D.E. Grobbee:** Writing – review & editing, Supervision, Conceptualization. **S.A.E. Peters:** Writing – review & editing, Supervision, Methodology, Funding acquisition, Conceptualization.

## Ethical statement

In this study, data from the PHARMO Data Network was used. This study was approved by the compliance committee of the PHARMO Institute.

## Funding

A VIDI fellowship was awarded by the Dutch Organization for Health Research and Development (ZonMW) (09150172010050) to SAEP.

## Declaration of competing interest

There are no conflicts of interests to declare.

## Data Availability

Data are available at PHARMO Institute for Drug Outcome Research upon reasonable request.
