# Supplementary material for: Sex differences in single and combined cardiovascular medication use
Source: Am Heart J Plus. 2026 Aug 22;70:100866. doi: 10.1016/j.ahjo.2026.100866 (PMC13543110; doi:10.1016/j.ahjo.2026.100866)
Supplement: Supplementary file 1 [file mmc1.docx]

**Supplementary material**

**Supplementary Table 1**. Categorization of CVDs and risk factors based on NHG-ICPC-1 codes

|  |  | **NHG-ICPC-1 codes** |
| --- | --- | --- |
| *Cardiovascular disease* | *Stroke* | K89, K90, K90.01, K90.02, K90.03 |
|  | *Arrhythmia* | K78, K79, K79.01, K79.02, K80, K80.01, K80.02, K80.03 |
|  | *Coronary Heart Disease* | K74, K74.01, K74.02, K75, K76, K76.01, K76.02 |
|  | *Peripheral Artery Disease* | K92, K92.01, K92.02, K92.03 |
|  | *Heart Failure* | K77, K77.01, K77.02 |
|  | *Other* | A89.01, K83, K83.01, K83.02, K83.03, K84.03, K99.01 |
| *Risk factor* | *Atherosclerosis* | K91 |
|  | *Hypertension* | K86, K87 |
|  | *Diabetes Mellitus* | T90, T90.01, T90.02 |
|  | *Chronic Kidney Disease* | U99.01 |

**Supplementary Table 2.** Ranges of plausible values for examination data.

| **Measurement data** | **Value range** |
| --- | --- |
| Systolic Blood Pressure | 40-300 mmHg |
| Diastolic Blood Pressure | 0-150 mmHg |
| Body Mass Index | 8-80 kg/m^2^ |
| Total Cholesterol | 0.5-20 mmol/L |
| High Density Lipoprotein | 0.5-20 mmol/L |
| Low Density Lipoprotein | 0.01-20 mmol/L |
| HbA1c | 20-200 mmol/mol |
| eGFR | 1-200 mL/min/1.73m^2^ |
| Creatinine | 1-200 μmol/L |

**Supplementary Table 3**. Categorization of cardiovascular drug categories based on their ATC code. Active substances are categorized in the ten main categories used in this paper, as well as global categories that are used in the baseline table.

| **Global drug category** | **Main drug category** | **ATC codes** |
| --- | --- | --- |
| Lipid-level modifiers | Other lipid-level modifiers | C10AB02, C10AB04, C10AB08,  C10AC01, C10AC04, C10AD02, C10AD06, C10AD52, C10AX06, C10AX09, C10AX13, C10AX14,  C10BA02, C10BA05, C10BA06,  C10BX01 |
|  | Statins | C10AA01, C10AA02, C10AA03, C10AA04, C10AA05, C10AA07,  C10BA02, C10BA05, C10BA06,  C10BX01 |
| Antithrombotics | Antithrombotics | B01AA03, B01AA04, B01AA07  B01AB01, B01AB04, B01AB05, B01AB06, B01AB09, B01AB10,  B01AC04, B01AC05, B01AC06, B01AC07, B01AC08, B01AC22, B01AC24, B01AC27, B01AC30, B01AC56, B01AE07, B01AF01, B01AF02, B01AF03, B01AX05 |
| Antihypertensives | ACE inhibitors (ACEi) | C09AA01, C09AA02, C09AA03, C09AA04, C09AA05, C09AA06, C09AA07, C09AA08, C09AA09, C09AA15, C09BA01, C09BA02, C09BA03, C09BA04, C09BA05, C09BA06, C09BA09, C09BB02, C09BB04, C09BB10, C09BX01, C09BX02 |
|  | Angiotensin II receptor blockers (ARBs) | C09CA01, C09CA02, C09CA03, C09CA04, C09CA06, C09CA07, C09CA08, C09DA01, C09DA02, C09DA03, C09DA04, C09DA06, C09DA07, C09DA08, C09DB01, C09DB02, C09DX01, C09DX03, C09DX04 |
|  | Calcium channel blockers (CCBs) | C08CA01, C08CA02, C08CA03, C08CA04, C08CA05, C08CA06, C08CA08, C08CA09, C08CA12, C08CA13, C08DA01, C08DB01,  C08GA02, C09BB02, C09BB04, C09BB10, C09BX01, C09DB01, C09DB02, C09DX01, X09DX03 |
|  | Beta blockers | C07AA02, C07AA03, C07AA05, C07AA07, C07AB02, C07AB03, C07AB04, C07AB05, C07AB07, C07AB08, C07AB12, C07AG01, C07AG02, C07BB02, C07BB07,  C07CA03, C07CB03, C09BX02 |
|  | Diuretics | C03AA03, C03AA04, C03CA01, C03CA02, C03DA01, C03DA04,  C03DB01, C03DB02, C03EA01, C03EA03, C07BB02, C07BB07,  C07CA03, C07CB03, C08GA02,  C09BA01, C09BA02, C09BA03, C09BA04, C09BA05, C09BA06, C09BA09, C09BX01, C09DA01, C09DA02, C09DA03, C09DA04, C09DA06, C09DA07, C09DA08,  C09DX01, C09DX03 |
| Other | Digoxin | C01AA05 |
|  | SGLT2 inhibitors (SGLT2i) | A10BK01, A10BK02, A10BK03, A10BK04 |

**Supplementary Table 4**. Scaled median women to men PRs (2010-2020) and [IQR] for the dispensed drug combinations, stratified by age and CVD status. Only the drug combinations that had an unscaled sex-combined prevalence of ≥1% are included.

| **Drug combination** | **n drug types** | **high-risk for CVD** | | **CVD diagnosed** | |
| --- | --- | --- | --- | --- | --- |
|  |  | **<70 years old** | **≥70 years old** | **<70 years old** | **≥70 years old** |
| ACEi | 1 | 0.6 [0.2] | 0.9 [0.1] |  |  |
| Antithrombotic | 1 |  | 0.9 [0.1] | 1.3 [0.1] | 1.0 [0.1] |
| ARB | 1 | 0.8 [0.3] | 1.0 [0.1] |  |  |
| Beta blocker | 1 | 1.3 [0.3] | 1.5 [0.2] | 2.4 [0.2] |  |
| CCB | 1 | 0.7 [0.3] | 1.2 [0.1] |  |  |
| Diuretic | 1 | 1.1 [0.4] | 1.8 [0.3] |  |  |
| Statin | 1 | 1.2 [0.1] | 0.7 [0.1] | 1.5 [0.2] | 1.2 [0.2] |
| ACEi - Diuretic | 2 | 0.7 [0.2] | 1.0 [0.1] |  |  |
| ACEi - Statin | 2 | 0.7 [0.2] | 0.7 [0.1] |  |  |
| Antithrombotic - Beta blocker | 2 |  | 1.1 [0.1] | 1.2 [0.1] | 1.2 [0.2] |
| Antithrombotic - Diuretic | 2 |  |  |  | 1.6 [0.4] |
| Antithrombotic - Statin | 2 | 1.1 [0.6] | 0.56 [0.0] | 0.9 [0.1] | 0.7 [0.1] |
| ARB - Diuretic | 2 | 0.8 [0.3] | 1.4 [0.3] |  |  |
| ARB - Statin | 2 | 1.0 [0.5] | 0.8 [0.1] |  |  |
| Beta blocker - Diuretic | 2 | 1.2 [0.5] | 2.5 [0.5] |  |  |
| Beta blocker - Statin | 2 | 1.6 [0.6] |  |  |  |
| Diuretic - Statin | 2 | 2.2 [0.8] | 1.4 [0.2] |  |  |
| ACEi - Antithrombotic - Beta blocker | 3 |  |  |  | 1.0 [0.3] |
| ACEi - Antithrombotic - Statin | 3 | 0.7 [0.4] | 0.5 [0.1] | 0.5 [0.2] | 0.5 [0.1] |
| ACEi - CCB - Statin | 3 | 0.7 [0.4] |  |  |  |
| ACEi - Diuretic - Statin | 3 | 1.0 [0.3] | 0.8 [0.1] |  |  |
| Antithrombotic - Beta blocker - Diuretic | 3 |  |  |  | 2.1 [0.1] |
| Antithrombotic - Beta blocker - Statin | 3 | 0.6 [0.2] | 0.5 [0.0] | 0.7 [0.1] | 0.6 [0.1] |
| Antithrombotic - CCB - Statin | 3 |  |  | 1.0 [0.2] | 0.9 [0.2] |
| Antithrombotic - Diuretic - Statin | 3 |  |  |  | 1.1 [0.3] |
| ARB - Antithrombotic - Statin | 3 |  |  | 0.8 [0.1] | 0.7 [0.1] |
| ARB - Diuretic - Statin | 3 | 1.1 [0.3] | 1.0 [0.1] |  |  |
| Beta blocker - Diuretic - Statin | 3 | 2.2 [0.8] |  |  |  |
| ACEi - Antithrombotic - Beta blocker - Diuretic | 4 |  |  |  | 1.4 [0.3] |
| ACEi - Antithrombotic - Beta blocker - Statin | 4 | 0.5 [0.3] | 0.4 [0.1] | 0.4 [0.0] | 0.5 [0.0] |
| ACEi - Antithrombotic - CCB - Statin | 4 |  |  | 0.6 [0.1] |  |
| ACEi - Antithrombotic - Diuretic - Statin | 4 |  | 0.6 [0.1] | 1.0 [0.1] | 0.7 [0.1] |
| ACEi - Beta blocker - Diuretic - Statin | 4 | 1.1 [0.5] |  |  |  |
| ACEi - CCB - Diuretic - Statin | 4 | 0.8 [0.3] |  |  |  |
| Antithrombotic - Beta blocker - CCB - Statin | 4 |  |  | 0.9 [0.2] | 0.8 [0.2] |
| Antithrombotic - Beta blocker - Diuretic - Statin | 4 |  | 1.2 [0.1] |  | 1.2 [0.1] |
| ARB - Antithrombotic - Beta blocker - Diuretic | 4 |  |  |  | 2.1 [0.3] |
| ARB - Antithrombotic - Beta blocker - Statin | 4 |  |  | 0.7 [0.1] | 0.7 [0.1] |
| ARB - Antithrombotic - Diuretic - Statin | 4 |  |  |  | 0.9 [0.2] |
| ARB - CCB - Diuretic - Statin | 4 | 0.7 [0.3] |  |  |  |
| ACEi - Antithrombotic - Beta blocker - CCB - Statin | 5 |  |  | 0.6 [0.1] | 0.5 [0.1] |
| ACEi - Antithrombotic - Beta blocker - Diuretic - Statin | 5 | 0.8 [0.4] | 0.6 [0.1] | 0.7 [0.1] | 0.7 [0.1] |
| ARB - Antithrombotic - Beta blocker - CCB - Statin | 5 |  |  |  | 0.9 [0.1] |
| ARB - Antithrombotic - Beta blocker - Diuretic - Statin | 5 |  | 1.0 [0.1] | 1.0 [0.1] | 1.0 [0.1] |
| ACEi - Antithrombotic - Beta blocker - CCB - Diuretic - Statin | 6 |  | 0.7 [0.2] | 0.7 [0.2] | 0.7 [0.1] |
| ARB - Antithrombotic - Beta blocker - CCB - Diuretic - Statin | 6 |  |  | 0.8 [0.1] | 1.0 [0.1] |

**Supplementary Figure 1.** Flowchart of in- and exclusion criteria for the study population.

^1^ This is the number of patients with a CVD diagnosis at baseline. It is allowed for patients to cross-over from the high-risk for CVD group to the CVD diagnosed group.

**Supplementary Figure 2.** Sample size per sex, age, and CVD status over the semesters from January 2010 to December 2020.

**Supplementary Figure 3.** Cumulative proportion of the number of units dispensed at a time. A maximum of 365 units per dispense moment was included in the graph for clarity.

**Supplementary Figure 4.** Flowchart of the statistical analysis.

**Supplementary Figure 5.** Prevalence of active substances over time (2010-2020) by sex, age, and CVD status.

**Supplementary Figure 6.** Median prevalence of single and combined dispensed drugs by sex and age in people diagnosed with CHD. Median prevalence was taken from 2010-2020. Error bars indicate the first and third quartile.
